# Areas of cerebral blood flow changes on arterial spin labelling with the use of symmetric template during nitroglycerin triggered cluster headache attacks

**DOI:** 10.1016/j.nicl.2021.102920

**Published:** 2021-12-22

**Authors:** Diana Y. Wei, Owen O'Daly, Fernando O. Zelaya, Peter J. Goadsby

**Affiliations:** a Headache Group, Wolfson Centre for Age-Related Diseases, King's College London, UK; b NIHR Wellcome Trust King's Clinical Research Facility, King's College Hospital, London, UK; c Centre for Neuroimaging Sciences, Department of Neuroimaging, Institute of Psychiatry, Psychology & Neuroscience, King's College London, London, UK

**Keywords:** Cluster headache, Arterial spin labelling, Functional MRI, Nitroglycerin, Symmetric template normalisation, Hypothalamus

## Abstract

**Background:**

Cluster headache is a rare, strictly unilateral, severe episodic primary headache disorder. Due to the unpredictable and episodic nature of the attacks, nitroglycerin has been used to trigger attacks for research purposes to further our understanding of cluster headache pathophysiology.

**Objectives:**

We aimed to identify regions of significant cerebral blood flow (CBF) changes during nitroglycerin triggered cluster headache attacks, using MRI with arterial spin labelling (ASL).

**Methods:**

Thirty-three subjects aged 18–60 years with episodic and chronic cluster headache were recruited and attended an open clinical screening visit without scanning to receive an intravenous nitroglycerin infusion (0.5 μg/kg/min over 20 min). Those for whom nitroglycerin successfully triggered a cluster headache attack, were invited to attend two subsequent scanning visits. They received either single-blinded intravenous nitroglycerin (0.5 μg/kg/min) or an equivalent volume of single-blinded intravenous 0.9% sodium chloride over a 20-minute infusion. Whole-brain CBF maps were acquired using a 3 Tesla MRI scanner pre-infusion and post-infusion. As cluster headache is a rare condition and purely unilateral disorder, an analysis strategy to ensure all the image data corresponded to symptomatology in the same hemisphere, without losing coherence across the group, was adopted. This consisted of spatially normalising all CBF maps to a standard symmetric reference template before flipping the images about the anterior-posterior axis for those CBF maps of subjects who experienced their headache in the right hemisphere. This procedure has been employed in previous studies and generated a group data set with expected features on the left hemisphere only.

**Results:**

Twenty-two subjects successfully responded to the nitroglycerin infusion and experienced triggered cluster headache attacks. A total of 20 subjects completed the placebo scanning visit, 20 completed the nitroglycerin scanning visit, and 18 subjects had completed both the nitroglycerin and placebo scanning visits. In a whole-brain analysis, we identified regions of significantly elevated CBF in the medial frontal gyrus, superior frontal gyrus, inferior frontal gyrus and cingulate gyrus, ipsilateral to attack side, in CBF maps acquired during cluster headache attack; compared with data from the placebo session. We also identified significantly reduced CBF in the precuneus, cuneus, superior parietal lobe and occipital lobe contralateral to the attack side. Of particular interest to this field of investigation, both the hypothalamus and ipsilateral ventral pons showed higher CBF in a separate region of interest analysis.

**Conclusion:**

Our data demonstrate that severe cluster headache leads to significant increases in regional cerebral perfusion, likely to reflect changes in neuronal activity in several regions of the brain, including the hypothalamus and the ventral pons. These data contribute to our understanding of cluster headache pathophysiology; and suggest that non-invasive ASL technology may be valuable in future mechanistic studies of this debilitating condition.

## Highlights

• Cluster headache is a severe unilateral primary headache disorder; however, the brain is asymmetric, therefore using a symmetric template before flipping in the x-axis allows for ipsilateral analysis of attacks without loss of coherence across the group.
• Increases in cerebral blood flow beyond pain anticipation, processing and modulation areas, including hypothalamic regions and ipsilateral pons, have a crucial pathophysiological role in cluster headache attacks.
• The pain experienced during cluster headache attacks is so severe that it “switches off” areas involved in the default brain network.

## 1 Introduction

Cluster headache occurs in approximately 0.1% of the population. It is a strictly unilateral primary headache; patients experience cluster headache attacks in a unilateral pattern in the trigeminal nerve distribution with ipsilateral cranial autonomic symptoms and restlessness (Headache Classification Committee of the International Headache Society (IHS), 2018). These attacks are severe, its intensity often compared to gunshot wounds, renal stones and childbirth (Burish et al., 2021). Although patients report attacks with a circadian pattern, spontaneous cluster headache attacks are difficult to predict and capture. Cluster headache attacks can be triggered by nitroglycerin (NTG) (Peters, 1953) and are comparable to spontaneous cluster headache attacks (Ekbom, 1968, Goadsby and Edvinsson, 1994, Fanciullacci et al., 1995), allowing researchers to study cluster headache attacks in a controlled and systematic way. Another challenge to imaging cluster headache attacks is that the disorder is rare. To reflect the underlying pathophysiology with is strictly lateralised pain, imaging studies in the past have flipped the image data accordingly (May et al., 1998, Sprenger et al., 2007, Magis et al., 2011, May et al., 1999, Absinta et al., 2012, Naegel et al., 2014, Arkink et al., 2017a, Arkink et al., 2017b, Yang et al., 2015, Chou et al., 2017, Faragó et al., 2017, Ferraro et al., 2018, Teepker et al., 2012, Szabó et al., 2013) in order to make the findings generalisable and compare all attack presentations as superimposed on only one side.

Hsieh and colleagues carried out the first study of cluster headache in humans using ^15^O labelled Positron Emission Tomography (PET) (Hsieh et al., 1996). They scanned acute cluster headache attacks after administration of 1 mg sublingual NTG in four episodic cluster headache patients, two patients with left-sided and two with right-sided attacks. In this small study where the images were not flipped, the authors found a significant increase in cerebral blood flow (CBF) in the right hemisphere in the caudal anterior cingulate caudate (ACC), and rostrocaudal ACC, tempororpolar region, supplementary motor area, bilateral primary motor area, premotor areas, opercular region, insula, putamen and lateral inferior frontal cortex. There were CBF reductions in the bilateral posterior parietal cortex, occipitotemporal region and prefrontal cortex. The subsequent H_2_^15^O PET study with nine chronic cluster headache patients used inhalation of NTG (1.0–1.2 mg) to trigger cluster headache attacks. Acknowledging the laterality of the condition, the authors mirrored some of the cluster headache attack images so that data corresponded to attacks on the left hemisphere. The authors found increases in CBF in several regions, including the bilateral ACC, contralateral posterior thalamus, ipsilateral basal ganglia, bilateral insulae and cerebellar hemispheres. Furthermore, an increase in regional CBF was found in the region of the posterior hypothalamic grey matter (Talairach coordinates −2, −18, −8) ipsilateral to attack side (May et al., 1998). Due to the episodic nature of cluster headache, only a handful of imaging studies have captured spontaneous cluster headache attacks (May et al., 2000, Sprenger et al., 2004, Morelli et al., 2009, Qiu, 2012, Morelli et al., 2013) and have found increases in CBF in the ipsilateral hypothalamic region22-24, (Morelli et al., 2013). Cluster headache is classified as a trigeminal autonomic cephalalgias (TAC) based on shared clinical characteristics with other primary unilateral headache disorders with ipsilateral cranial autonomic symptoms. It is believed that TACs share underlying pathophysiology and in particular the neuroimaging findings indicating the importance of the hypothalamic region. This has been found in short-lasting unilateral neuralgiform headache attacks with conjunctival injection and tearing (SUNCT) (May et al., 1999, Sprenger et al., 2005, Cohen, 2007) and in paroxysmal hemicrania (Matharu et al., 2006).

NTG is a precursor of nitric oxide, and it is known to trigger cluster headache attacks when patients are ‘in bout’, indicating the period when patients have repeated spontaneous cluster headache attacks. However, when patients are ‘out of bout’, they do not experience spontaneous attacks, and NTG does not trigger attacks during this phase. NTG can bring on an immediate mild and generalised ‘NTG-induced headache’, a separate and distinctly different headache than the delayed cluster headache attacks in patients (Ekbom, 1968, Wei and Goadsby, 2021). The neuroimaging effects of NTG were demonstrated in a H_2_^15^O PET study of eight episodic cluster headache patients, carried out in the out-of-bout phase, with 1–1.2 mg NTG inhalation. This group of patients did not have cluster headache attacks; however, they did develop a mild NTG headache. When comparing NTG headache with rest, there were increases in signal bilaterally in the anterior cingulate, right posterior thalamus, left basal ganglia, both frontal lobes, bilateral insulae and left temporal lobe (May et al., 2000). The same study compared nine chronic cluster headache subjects at rest and after the NTG spray, but before the onset of cluster headache attacks. This showed increased signal in the large intracranial vessels. Similarly, in a H_2_^15^O PET study with 24 migraine patients who received 0.5 μg/kg/minute over 20 min, they found that during the NTG headache, there was no signal increase in the dorsal pons, which was present in NTG triggered migraine attacks; however there were increases in signal in the anterior cingulate and corresponding regions to the internal carotid and basilar arteries (Afridi, 2005).

In the study of several pain conditions including cluster headache, ASL offers important benefits compared with H_2_^15^O PET and blood-oxygen-level-dependent (BOLD) functional MRI. H_2_^15^O PET has a low spatiotemporal resolution and the positron emitting nucleus (^15^O) has a half-life of approximately 2 min. PET is invasive, and requires the use of a radioactive tracer, exposing subjects to ionising radiation, limiting the number of its applications. ASL on the other hand uses magnetically labelled water within arterial blood as the endogenous tracer, (Detre et al., 1992, Alsop et al., 2015) to measure regional CBF; therefore, it is therefore entirely non-invasive. As subjects are not exposed to ionising radiation, subjects can safely have repeated scans and repeated visits, beneficial in the study of this type of repeated measures pain study designs. ASL also allows for quantitative measurements of CBF, whereby, as a result of neuro-vascular coupling, changes in the magnitude of CBF serve as an indirect but sensitive marker of neuronal activity changes. Neuronal activity requires increased energy demands; this energy demand is supplied from functional hyperaemia. Neurovascular coupling is the phenomenon that transiently links changes in neural activity with localised increases in CBF.

Since cluster headache is a strictly unilateral condition, rotation of image data is performed to reflect the underlying pathophysiology. However, most human brains are not symmetric in morphology (Toga and Thompson, 2003, Watkins, 2001), reference brain templates commonly used in neuroimaging also reflect this asymmetry. Consequently, flipping of the image data after normalising the data to those templates about any given axis can lead to significant errors of spatial alignment of the images. To eliminate this limitation, studies performing interhemispheric comparisons normalise to a symmetrical template first. A symmetrical template can be generated in two ways. The first method is by flipping subject images left–right, once normalised to an asymmetric template, averaging all the images treating the reversed subjects as new additional subjects (Watkins, 2001, Beraha et al., 2012, Hougaard et al., 2015). The second method is to flip the asymmetric template and to average with the non-flipped atlas (Baciu et al., 2005, Hougaard et al., 2015). Some studies used a premade symmetric template (Lo et al., 2010, Vallesi et al., 2017, Kosztyla et al., 2016, Grabner, 2006). In this study, we made use of a ‘Symmetric Brain Template’ from the Montreal Neurological Institute (Fonov et al., 2009, Fonov et al., 2011). Therefore, before flipping our data about the anterior-posterior axis, our images were normalised to this symmetric template, which allowed us to analyse the images as if they corresponded to unilateral presentation in the same hemisphere. This strategy also addressed the complications generated by the natural asymmetries of the human brain.

This is the first study to use ASL to investigate cluster headache attacks, and the first study we are aware of, to exploit the availability of a symmetric reference template to normalise spatially the image data, to analyse the images as corresponding to unilateral cluster headache attacks. A recent study used ASL to investigate the interictal effects of greater occipital nerve block in the treatment in cluster headache (Medina et al., 2021).

## 2 Material and methods

### 2.1 Subject selection and recruitment

The study was advertised in the UK cluster headache patient website OUCH (UK) (Organisation for the Understanding of Cluster Headache; https://ouchuk.org/research/research-volunteers-needed and the tertiary Headache Centre in King's College Hospital, London. Patients interested in participating contacted the investigating team via a dedicated research email address. Patients would then be screened for eligibility for the study via emails and a telephone call; those who met the criteria were invited to attend a screening visit. Data were collected between January 2017 until January 2019.

Recruitment was limited to subjects between the ages of 18 and 60 with cluster headache according to the ICHD-3 beta criteria (Headache Classification Committee of the International Headache Society IHS, 2013), no previous syncope or history of autonomic dysfunction, reliable response to high flow oxygen and/or subcutaneous sumatriptan during spontaneous attacks and previous normal structural neuroimaging. Women of childbearing age were required to use reliable contraceptive methods during the study. Subjects were excluded if there were any contraindications to MRI scan, those who were pregnant or breastfeeding, any significant psychiatric disease, diagnosis of another primary headache type (other than migraine) or chronic pain syndrome, any medical history that would have contraindications to receiving NTG, preventive medication other than verapamil, if taking indomethacin for any reason, if they had allergies to the medications used in the study or intolerance to high flow oxygen and if they used of illicit drugs for six months before and during the study.

All subjects gave written informed consent to participate. The study received the National Research Ethics Service approval (16/LO/0693).

### 2.2 Study protocol

The study was comprised of three study visits, with each visit separated by a minimum of one week. Each subject underwent a full headache consultation during the first visit detailing their headache phenotype, medical history, general physical examination, and neurological examination. Each subject had an electrocardiogram and supine and standing blood pressure measurement, oxygen saturation measurement, and recording of pulse and weight. All female subjects were required to have a negative urine pregnancy test before the start of the infusion.

#### 2.2.1 Open NTG infusion visit

If deemed eligible, subjects received an NTG infusion intravenously and were not scanned in this visit. Subjects remained recumbent for 30 min before the infusion and had their blood pressure and pulse checked before the infusion. The infusion rate was calculated based on a weight-adjusted dose of 0.5 μg/kg/min over 20 min. This session had the objective of establishing if a cluster headache could be triggered in each subject after the administration of NTG.

Every five minutes, subjects were asked to rate their pain level, report the presence of cranial autonomic symptoms and non-headache symptoms (see supplementary material), and their blood pressure and pulse rate were also measured. After the NTG infusion, subjects received 250 mL of 0.9% sodium chloride solution intravenously. Acute cluster headache treatment was administered at twenty minutes from the start of attack either with sumatriptan 6 mg subcutaneous injection or with 15 L/min oxygen via a non-rebreather mask, as standard cluster headache attack treatment. The visit concluded only when the subject was pain-free. Subjects were asked to keep a headache diary and note if they developed a headache later that day, which was out of their regular pattern.

#### 2.2.2 Scanning visits

After administration of NTG, subjects who were successfully triggered with a cluster headache attack were invited to return for the two scanning visits. All scans occurred at similar period of day, between 9 am and 1 pm, to minimise known circadian rhythm effects on regional cerebral blood flow (Hodkinson et al., 2014). Subjects were asked to have a light breakfast, remain fasting throughout the visit, and avoid caffeine 12 h before the visit. Subjects had one scanning visit in which they received intravenous NTG at 0.5 μg/kg/min over 20 min and another one in which they received the equal volume of 0.9% sodium chloride, at the same rate of infusion of NTG, over 20 min. If there were significant movement artefacts on the images, subjects were invited to return for another scanning visit.

### 2.3 Magnetic resonance imaging acquisition

Subjects were scanned in the supine position on a 3 T General Electric Discovery MR750 MRI scanner using a 32-channel head coil. Subjects were scanned twice in each visit; the first pre-infusion scan was called the “baseline” scan, which included structural and functional images, and the second scan post-infusion was called the “triggered attack” scan in the NTG visit and “post-infusion” scan in the placebo visit, this included only the functional images. The NTG “triggered attack” scan was acquired from the start of the attack. The start of the attack is defined as unilateral pain in the trigeminal distribution with either CAS and or sense of agitation, and the subject felt this was comparable to their spontaneous attacks. During the placebo visit, the “post-infusion” scan was performed at the time when an attack was experienced from the unblinded NTG non-scanning visit.

High-resolution 3D T1-weighted sagittal MPRAGE (Magnetisation Prepared Rapid Acquisition Gradient Echo) images were acquired to facilitate the spatial normalisation of the CBF maps. The parameters of this scan were TR = 7.312 ms; TE = 3.016 ms; FOV = 270 mm; matrix = 256x256mm; slice thickness = 1.2 mm; 196 slice partitions. T2 images were acquired using a 2D Multi Slice Fast Spin Echo protocol, with the following parameters: TR = 4380 ms; TE = 54.84 ms; FOV = 240 mm; matrix = 320 × 256; slice thickness = 2 mm with 0 mm spacing; 72 slice partitions. Only the T1 weighted images were used in pre-processing and spatial normalisation of the data.

Whole-brain CBF maps were generated using a 3D pseudo-continuous Arterial Spin Labelling (3D-pCASL) MRI sequence. This ASL modality is known to provide a higher signal-to-noise ratio compared with other methods (Dai et al., 2008). This ASL variant has the advantage of a highly efficient train of short (500 µs) pulses of radiofrequency and gradient fields, which are more compatible with clinical body coil transmission hardware and have become the method of choice for *in vivo* cerebral perfusion investigations (Alsop et al., 2015).

Labelling of arterial blood was achieved with a 1825 ms train of Hanning shaped RF pulses of 500 µs duration in the presence of a net magnetic field gradient along the flow direction (the z-axis of the magnet). After a post-labelling delay of 2025 ms, a whole-brain volume was read using a 3D inter-leaved “stack-of-spirals” Fast Spin Echo readout, consisting of 8 interleaved spiral arms in the in-plane direction, with 512 points per spiral interleave. The images had 60 axial slice locations (3 mm thickness) and an in-plane FOV of 240 × 240 mm after transformation to a rectangular matrix (TE/TR = 11.088/5180 ms, flip angle (FA) = 111°). A proton density (PD) image volume with the same parameters was acquired within the same sequence parameters in the readout, to use as a reference to compute the CBF maps in conventional physiological units (mL blood/100 g tissue/minute). Four control-labelled pairs of images were acquired in each run. The total acquisition time was 6:08 min.

The sequence used four background suppression pulses to minimise static tissue signal at the time of image acquisition. Two ASL scans of 6:08 min duration were acquired during the “baseline”, the “triggered attack” and ”post-infusion” scan sessions. During the “triggered attack” scan, subjects had the option to stop the scan if their level of agitation during their attack was too severe to stay still in the scanner; therefore, in some cases, one CBF map was acquired. CBF maps were computed from the mean perfusion-weighted difference image derived from the two control-label pairs by scaling the difference image against a proton density image acquired at the end of the sequence, using identical readout parameters.

### 2.4 Imaging pre-processing

Pre-processing of pCASL images (spatial normalisation) was performed using Automated Software for ASL Processing (ASAP) toolbox (Mato Abad et al., 2016) which employs the Statistical Parametric Mapping software suite, version 12 (SPM 12; www.fil.ion.ucl.ac.uk/spm/). Voxel-wise computation of CBF was performed by the scanner software, using the formula recommended by the ASL consensus article (Alsop et al., 2015):

$$\mathrm{CBF}=6000\frac{e^{w/T_{1a}}}{2\varepsilon T_{1a}(\left(1-e^{-\tau /T_{1a}}\right)}\frac{P\lambda }{R}$$

In which P is the signal in the averaged perfusion-weighted image (control–label), R is the signal in the reference (proton density) image, ε is the combined efficiency of labelling and background suppression (∼65%), τ is the label duration (1825 ms), $T_{1a}$ is the $T_{1}$ of arterial water, and w is the post labelling delay (2025 ms), and λ is the scale factor for brain/blood partition coefficient in mL/g.

**Fig. 1:**
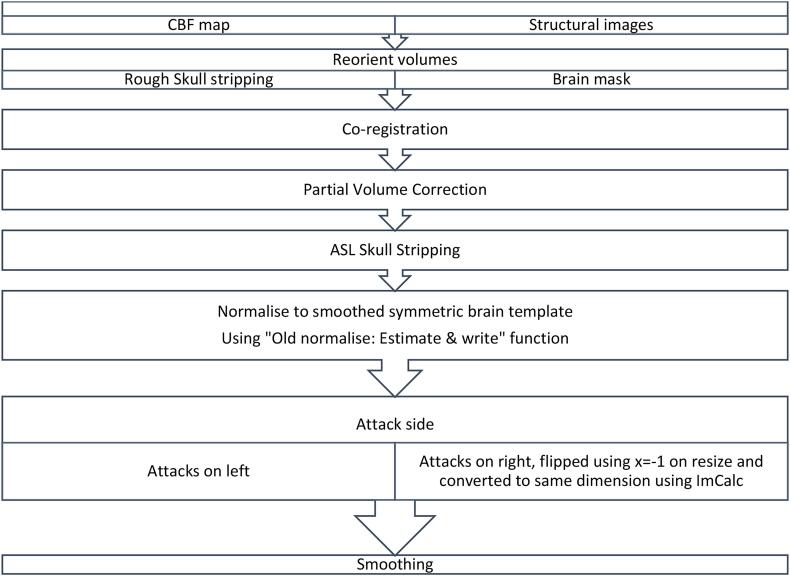
Normalisation of ASL data to a symmetric template.

A multistep approach was used for spatial normalisation of the CBF maps to the Symmetric space of the Montreal Neurological Institute (MNI) within the ASAP framework. CBF maps were co-registered to the high-resolution T1-weighted structural ADNI images after coarse alignment of the origin of both images. Unified segmentation of the T1-weighted image normalised this image to the MNI space and was used to produce a 'brain-only' binary mask which was multiplied by the co-registered rCBF map to produce an image free of extracerebral artefacts (Mato Abad et al., 2016) (Fig. 1). Each CBF map was reviewed and checked for quality of spatial normalisation against the symmetric brain template in SPM. Any data with significant movement artefact was excluded from the study. Each pair of CBF maps acquired at baseline or during headache was averaged into a single image after spatial normalisation.

### 2.5 Transformation of the normalised images to the symmetric MNI template

Based on the hypothesis that unilateral headache would lead mainly to ipsilateral CBF changes, we decided to perform a mirror-inversion (i.e flip) of the images about the anterior–posterior direction (in our case, all right-sided attacks were flipped to the left). As explained earlier, since human brains are not symmetric, our first step was to transform the normalised maps to a symmetric template, which is also available from the MNI software suite.

Once in a symmetric frame of reference, the images could be flipped' from right to left without losing coherence across the group. The symmetric template used was the ICBM 152 Nonlinear symmetric brain template (mni_icbm152_t1_tal_nlin_sym_09a.nii: http://www.bic.mni.mcgill.ca/ServicesAtlases/ICBM152NLin2009).

The side of the provoked attack was identified in each subject; all subjects with right-sided attacks had their images flipped along the x-axis to reflect all attacks on the left. Images were reoriented, resized x = -1 and then were smoothed using an 8x8x8mm Gaussian kernel. For those subjects who experienced headache on the left side, their data was normalised to the symmetric template but not flipped (Fig. 1).

### 2.6 Analysis of ASL data

The ASL data were analysed using a voxel-wise general linear model in SPM 12. Hypothesis-led region of interest (ROI) analysis was performed. Pre-determined masks were used to investigate the ROI significance; the masks were generated by the Wake Forest University School of Medicine (WFU) PickAtlas. The masks included separate right and left hemispheric: amygdala, anterior cingulate cortex, insula, substantia nigra, thalamus, hypothalamus and bilateral pons. These are areas were chosen given their involvement in pain and more specifically in cluster headache from previously published findings. Furthermore, we investigated the post hypothalamic region reported in the study by May and colleagues (May et al., 1998) at Talairach coordinates (-2, −18, −8). This location was converted to MNI coordinates using the WFU PickAtlas and found to be (-2,-18, −10.5).

Further, exploratory whole-brain voxel-wise flexible factorial, 2x2 ANOVA analysis was performed to assess CBF related changes when comparing placebo “baseline”, placebo “post-infusion”, and NTG “baseline”, NTG “triggered attack” scans. Furthermore, a whole-brain voxel-wise paired *T*-test allowed analysis of within-session changes in CBF related to NTG “baseline” with NTG “triggered attack” and placebo “baseline” with placebo “post-infusion” scans. Clusters of significant change were determined using the cluster-extent criterion (P_FWE_ < 0.05) using an uncorrected voxel-wise cluster-forming threshold of *P* < 0.005. Both whole-brain analyses included mean global CBF as a covariate in the design matrix using ANCOVA to account for inter-individual differences in global perfusion. All brain locations are reported as x, y, and z coordinates in Montreal Neurologic Institute (MNI) space.

### 2.7 Statistical analysis of demographics

Descriptive statistics and between-group differences were performed using SPSS Statistics version 26 for Mac and Excel for Mac v16.30, *p <* 0.05 was considered significant.

### 2.8 Sample size calculation

The sample size was calculated from the ASL based studies (Murphy et al., 2011), where approximately 16 subjects are needed to observe a significant change of 5 mL blood/100 g tissue/minute. Based on normative CBF data sets collected in many ASL based studies, we have been able to ascertain that with a standard deviation of 10% (from a grey matter mean of 5 mL blood/100 g tissue/minute), a minimum of 16 subjects are needed to observe a change of 5 mL blood/100 g tissue/minute (effect size 0.8 using G*Power), for a two-tailed, independent sample *T*-Test comparison.

## 3 Results

### 3.1 Demographics

**Fig. 2:**
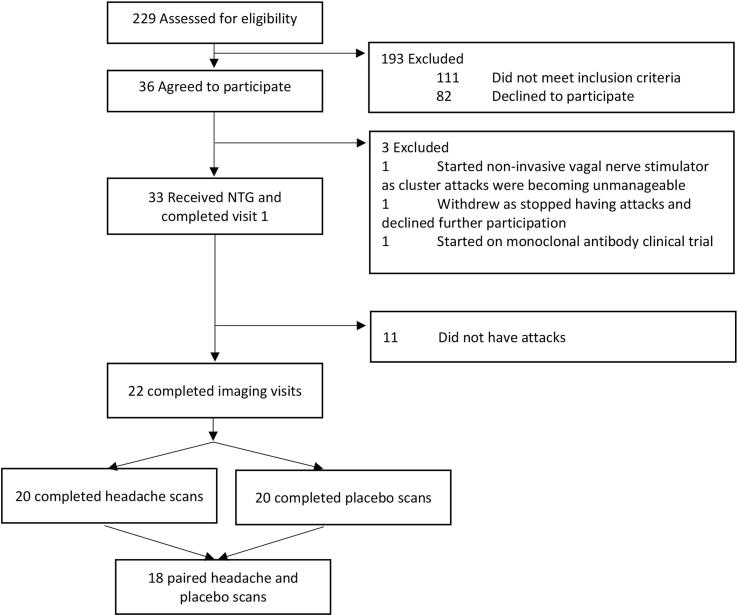
Subject numbers throughout the study.

A total of 229 subjects contacted us and were checked for eligibility, of which 33 were recruited into the study and attended the first visit. A cluster headache attack was successfully triggered in 22 subjects, and these individuals were included in the study. Ultimately, 20 participants completed the placebo visit and 20 completed the NTG visit, with the result that we had 18 subjects successfully scanned on both placebo and NTG visits (Fig. 2). There was an equal number of episodic compared with chronic cluster headache and an equal number of left-sided and right-sided attacks within the cohort. There were 15 male subjects (68%) and seven female subjects (32%). The mean average number of attacks per day was 2.6 (SD 1.8, range 0.3–7), and the median duration of untreated attacks was 82.5 min (IQR 45–150). A total of seven subjects used verapamil for cluster headache prevention (Table 1).

**Table 1:** Demographics and characteristics of study subjects. MWA = migraine with aura, MWoA = migraine without aura, TAWH = typical aura without headache,

Subject	Age	Handedness	Gender	Subtype	Side of attack	Years since first attack	Average attack frequency (per day)	Average duration of attacks when untreated (minutes)	Average bout duration (weeks)	Average bout frequency per year	Verapamil (daily total dose in mg)	Regular medications	Migraine history
1	55	Right	M	Chronic	Right	5	2	45	**–**	**–**	480	Omeprazole, simvastatin	No
2	28	Right	F	Chronic	Right	12	7	150	**–**	**–**	–	–	MWoA
3	40	Right	M	Episodic	Right	20	3	150	8	1	–	Ventolin inhaler	No
4	53	Right	M	Chronic	Left	15	4	35	**–**	**–**	960	Sertraline	No
5	43	Right	M	Chronic	Right	9	1	40	**–**	**–**	–	–	No
6	44	Right	M	Episodic	Right	27	6	45	6	2	–	Lansoprazole, nifedipine,salbutamol inhaler	TAWH
7	47	Left	M	Episodic	Right	3	1	105	20	1	–	–	No
8	35	Right	M	Episodic	Left	7	2	150	6	1	240	–	MWoA
9	58	Right	M	Episodic	Left	3	1	135	24	0.67	–	Ramipril, omeprazole,Betahistine	MWA
10	43	Right	M	Chronic	Left	4	2	60	–	–	–	–	MWA
11	32	Right	M	Episodic	Left	3	3	50	12	2	600	Atorvastatin, omeprazole, codeine	MWoA
12	49	Right	F	Episodic	Right	8	5	50	9	2	–	–	MWoA
13	49	Right	M	Chronic	Right	35	2	90	–	–	–	–	MWoA
14	23	Right	F	Episodic	Right	9	2.5	90	6	1	–	Low-oestrogen pill	MWoA
15	40	Right	M	Chronic	Left	17	1.5	180	–	–	–	Sertraline, lansoprazole, melatonin	MWoA
16	20	Right	F	Chronic	Right	6	1.5	150	–	–	–	–	No
17	31	Right	F	Episodic	Right	14	2	75	11	1	–	–	MWoA
18	35	Right	M	Episodic	Left	9	2	90	3	0.5	–	Vitamin D, Seretide, clenil and salbutamol inhaler	No
19	38	Right	F	Chronic	Left	6	1.5	180	–	–	240	–	MWoA
20	49	Right	M	Episodic	Left	0.5	1.5	37.5	21	1	400	–	MWA
21	35	Right	F	Chronic	Left	4	0.3	30	–	–	600	Salbutamol, fostair inhalers	No
22	32	Left	M	Chronic	Left	10	6	60	–	–	–	Omeprazole	MWoA

### 3.2 Experience of triggered attacks

The triggered attacks were comparable to the spontaneous attacks experienced by the subjects (Table 2). Five subjects opted to have just one pCASL scan during their attack on the NTG scanning visit, one subject had a repeated visit due to movement and one subject had a repeated visit as they were unable to tolerate headache scan at the first visit. The following subjects had repeated visits as two subjects developed migraine, one subject developed visual aura during their NTG-triggered cluster headache attack and three subjects developed spontaneous cluster headache attacks during their placebo visits (Table 2).

**Table 2:** Overview of the attacks experienced by subjects, comparing their spontaneous attacks with their experience during the open NTG visit, NTG scanning visit and placebo scanning visit.




The greyed-out boxes indicate scans that were not used in analysis, please refer to the comments column for explanation and detail. A = agitation, Au = aural fullness, CAS = cranial autonomic symptoms, C = conjunctival injection, E = eye grittiness, F = facial flushing, Fa = facial droop, Fs = facial swelling, L = lacrimation, M = miosis, N = nasal congestion, O_2_ = 15 L/min oxygen via non-rebreather mask, P = ptosis, pCASL = pseudo-continuous Arterial Spin Labelling, Pe = periorbital oedema, R = rhinorrhoea, S = sialorrhea, Suma = sumatriptan, T = throat swelling, V = voice change.

### 3.3 Flexible factorial analysis

#### 3.3.1 Whole-brain comparison

**Fig. 3:**
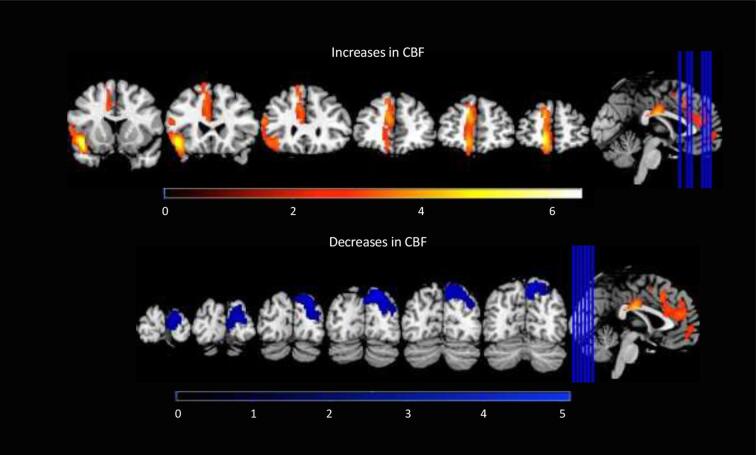
Coronal views of increases and decreases in CBF during cluster headache attack compared to baseline following intravenous nitroglycerin infusion with placebo headache with placebo baseline, following region of interest and flexible factorial analysis. Bars represent T-values.

Using a 2x2 factorial analysis to compare the four conditions of our study (placebo “baseline”, placebo “post-infusion” with NTG “baseline”, NTG “triggered attack” , *n* = 18), we identified two clusters of significantly increased in CBF in the NTG visit compared to placebo visit, the first cluster included the left medial frontal gyrus, left anterior cingulate gyrus, left superior frontal gyrus and right medial frontal gyrus. The second cluster included the left inferior frontal gyrus and the left precentral gyrus (Fig. 3) with the corresponding coordinates (Table 3). Significant reduction in CBF was also observed in the right precuneus, right cuneus, right superior parietal lobule, right occipital gyrus, right superior occipital gyrus and right middle occipital gyrus (Table 4).

**Table 3:** Brain regions of rCBF increases from flexible factorial analysis (*n* = 18), shown in coordinates in MNI space with relative T scores and k values.

Brain region Cluster description	*p*	Cluster size	Hemisphere	*T*	Peak coordinates		
		(k)			x	y	z
Medial frontal gyrus	0.000	3659	Left	5.76	−10	56	−6
			Left	4.28	−8	48	28
			Left	4.19	−6	46	20
			Left	3.98	−10	40	44
			Left	3.72	−8	26	50
			Left	3.52	−6	−10	52
			Left	3.46	−8	28	34
Cingulate gyrus			Left	4.57	−4	−16	28
			Left	3.97	−4	−14	36
			Left	3.92	−12	6	44
			Left	3.64	−6	20	38
			Left	3.63	−12	20	36
			Left	3.51	−12	18	30
			Left	3.46	−12	10	34
Superior frontal gyrus			Left	3.63	−10	26	54
Medial frontal gyrus			Right	3.79	18	66	−2
Inferior frontal gyrus	0.021	1286	Left	6.47	−44	18	−10
			Left	3.73	−54	28	16
			Left	3.05	−56	30	4
Precentral gyrus			Left	3.35	−60	10	12

**Table 4:** Brain regions of rCBF decreases with flexible factorial analysis (*n* = 18), shown in coordinates in MNI space with relative T scores and k values.

Brain region Cluster description	*p*	Cluster size	Hemisphere	*T*	Peak coordinates		
		(k)			x	y	z
Precuneus	0.000	3320	Right	5.10	20	−80	44
			Right	4.53	8	−66	52
			Right	4.47	10	−64	48
			Right	4.40	10	−72	52
			Right	3.36	14	−62	26
			Right	3.20	16	−52	58
Cuneus			Right	4.36	26	−84	32
			Right	3.98	16	−96	14
			Right	3.25	16	−100	−2
Superior parietal lobule			Right	3.85	30	−58	58
			Right	3.84	22	−70	58
			Right	2.85	32	−66	48
Occipital gyrus			Right	3.77	22	−90	14
Superior occipital gyrus			Right	3.40	40	−80	26
Middle occipital gyrus			Right	2.75	36	−86	8

#### 3.3.2 Region of interest analysis

**Fig. 4:**
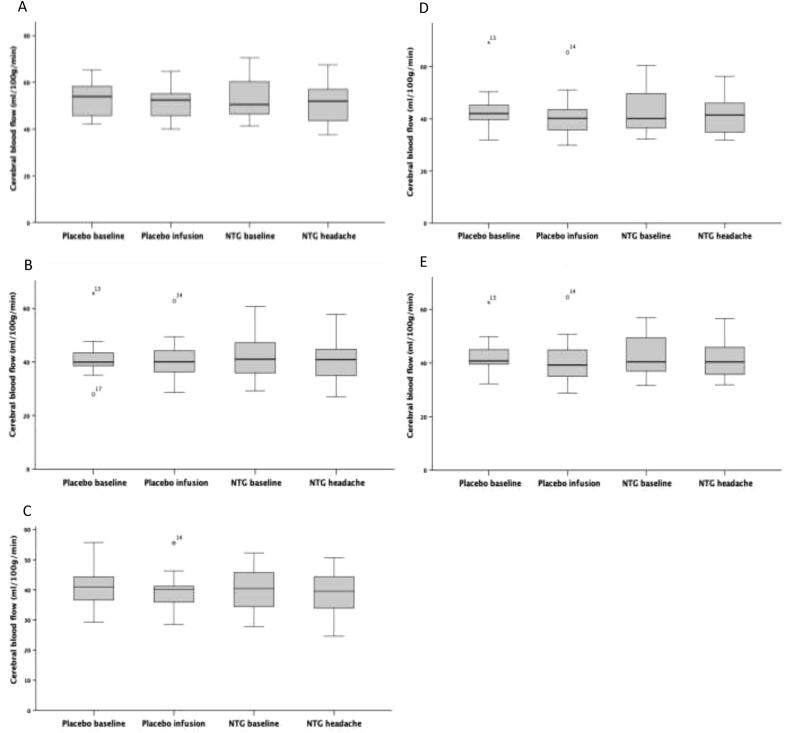
Boxplots depicting the change in mean cerebral blood flow (CBF) during each condition for regions found to show significant increases in CBF between the two visit days (placebo and NTG) in the left ACC (A), left thalamus (B), left pons (C), left hypothalamus (D) and right hypothalamus (E) with region of interest analysis using flexible factorial (*n* = 18).

Using a priori regions of interest, areas of significant increases in CBF were found in the left anterior cingulate cortex, bilateral hypothalamic regions, left thalamus and left pons (Table 5) with the changes in mean CBF values during each condition (Fig. 4).

**Table 5:** Regions of interest of increased rCBF with flexible factorial analysis (*n* = 18), with peak level P_FWE_ shown with corresponding coordinates in MNI space_._

Brain region Region of interest	Hemisphere	Peak level P_FWE_	Peak coordinates		
			x	y	z
ACC	Left	0.014	−6	44	16
	Left	0.018	−12	50	−4
	Left	0.018	−10	52	2
	Left	0.020	−14	18	26
	Left	0.022	−6	40	20
	Left	0.030	−6	56	2
Hypothalamus	Left	0.007	−8	−6	−4
	Right	0.010	4	−4	12
Thalamus	Left	0.007	−6	−18	18
	Left	0.027	−8	−22	16
Pons	Left	0.017	−2	−20	−38

### 3.4 Paired T-test comparing within session scans (“triggered attack” vs “baseline”)

#### 3.4.1 Whole-brain comparison NTG visits

Using paired T-test to compare NTG “triggered attack” with NTG “baseline” (*n* = 20), we determined two clusters of increased regional CBF, the first cluster included the left medial frontal gyrus, left anterior cingulate, left cingulate gyrus and left superior frontal gyrus. The second cluster included the left medial frontal gyrus, superior frontal gyrus, right superior frontal gyrus and right medial frontal gyrus (Table 6). There were two clusters of decreased regional CBF of statistical significance; the first included the right cuneus, right precuneus, right middle occipital lobe, right superior parietal lobe and right inferior occipital gyrus. The second cluster of decreased regional CBF included the left lingual gyrus, left superior occipital gyrus and left middle occipital gyrus, left cuneus, left sub-gyral and left precuneus regions (Table 7).

**Table 6:** Brain regions of rCBF increase in headache compared with baseline with paired T-test (*n* = 20) using symmetric and flipped maps, shown in coordinates in MNI space with relative T scores and k values.

Brain region Cluster description	*p*	Cluster size (k)	Hemisphere	*T*	Peak coordinates		
					x	y	z
Medial frontal gyrus	0.015	1072	Left	4.94	−8	42	20
			Left	4.75	−10	42	24
			Left	4.46	−8	56	0
			Left	3.88	−6	48	6
			Left	3.62	−6	18	52
			Left	3.48	−2	34	40
Anterior cingulate			Left	4.26	−6	34	24
Cingulate gyrus			Left	3.84	−6	20	42
			Left	3.61	−2	18	40
			Left	3.39	−10	6	44
Superior frontal gyrus			Left	3.57	−4	26	56
			Left	3.50	−4	28	52
Medial frontal gyrus	0.016	1052	Left	4.68	−6	66	−14
Superior frontal gyrus			Left	4.23	−8	68	−10
			Left	4.21	−18	70	0
			Left	4.17	−26	66	−4
			Left	4.15	−24	68	0
			Left	4.15	−22	68	−4
			Left	3.25	−30	54	28
			Left	3.15	−30	64	−8
			Left	3.10	−28	56	22
Superior frontal gyrus			Right	3.32	32	60	−16
			Right	3.31	14	68	−12
			Right	3.29	14	70	−8
Medial frontal gyrus			Right	3.16	18	68	−2

**Table 7:** Brain regions of rCBF decrease in headache compared with baseline using paired T-test (*n* = 20) with symmetric and flipped maps, shown in coordinates in MNI space with relative T scores and k values.

Brain region Cluster description	*p*	Cluster size (k)	Hemisphere	*T*	Peak coordinates		
					x	y	z
Cuneus	0.000	2638	Right	5.67	24	−92	10
			Right	4.92	28	−86	30
			Right	4.65	14	−98	14
			Right	3.91	8	−92	20
Precuneus			Right	4.81	20	−72	40
			Right	4.66	18	−70	50
			Right	4.40	16	−62	50
			Right	4.39	20	−58	58
			Right	3.55	16	−66	32
Middle occipital lobe			Right	4.60	26	−88	14
			Right	4.29	42	−84	2
			Right	3.95	40	−82	10
			Right	3.92	38	−86	6
Superior parietal lobe			Right	4.22	26	−60	46
			Right	4.12	22	−54	60
Inferior occipital gyrus			Right	4.87	40	−86	−6
Lingual gyrus	0.001	1678	Left	5.07	−14	−94	−4
			Left	5.01	−18	98	0
Superior occipital gyrus			Left	4.64	−34	−84	26
			Left	4.54	−34	−82	30
Middle occipital gyrus			Left	4.49	−36	−88	4
			Left	4.38	−36	−86	10
			Left	3.95	−32	−84	16
			Left	2.99	−48	−76	4
Cuneus			Left	4.29	−16	−92	26
			Left	4.17	−20	−88	32
			Left	4.12	−16	−88	34
							
Sub-gyral			Left	3.65	−26	−70	24
Precuneus			Left	3.16	−30	−70	34
			Left	3.16	−28	−64	36
			Left	3.05	−26	−68	40
			Left	3.02	−26	−72	38

#### 3.4.2 Region of interest comparison of within session scans

Using a priori selected regions, we demonstrated an increase in CBF when comparing NTG “triggered attack” with NTG “baseline” in the left posterior hypothalamus, left anterior cingulate and left substantia nigra (Table 8).

**Table 8:** Regions of interest of increased rCBF in headache compared with baseline using symmetric and flipped maps with paired T-test (*n* = 20), shown in MNI space with relative peak level P_FWE_ values.

Brain region Region of interest	Hemisphere	Peak level P_FWE_	Peak coordinates		
			x	y	z
Posterior hypothalamus	Left	0.021	−10	−18	−10
*8 mm sphere at −2,-18,-10.5*	Left	0.024	−6	−18	−12
	Left	0.027	−2	−16	−16
ACC	Left	0.024	−8	38	22
	Left	0.040	−14	36	22
Substantia nigra	Left	0.006	−10	−20	−10
	Left	0.017	−14	−20	−6

#### 3.4.3 Whole-brain comparison in images from the placebo visits

Using paired T-test to compare “headache” and “baseline” scans during the placebo visits sessions (*n* = 20), there were no areas of significance.

## 4 Discussion

Our data show that cluster headache attacks triggered by NTG are associated with increased CBF in areas involved in pain perception on the ipsilateral side to the attack, with increased CBF in the hypothalamus during an acute attack that was not present during the placebo visit. Our results are consistent with previous imaging studies into acute cluster headache attacks. By utilising the innovative method of normalising to a symmetric template before rotating the images, we could reliably analyse the image data without the complications caused by conventional using non-symmetric templates and increase our statistical power of detection of changes. This method proved to be a useful strategy in investigating this rare primary headache disorder, with a challenging study protocol.

The use of a symmetric template may be advantageous in other conditions where there is a clear lateralised aspect, such as in other rare primary unilateral headache disorders in the TAC group. Our most striking result is the lateralised and ipsilateral CBF changes during cluster headache attacks compared with placebo. The clusters of increased CBF in our analysis involved discrete peaks in the left inferior frontal gyrus, left medial frontal gyrus and left cingulate gyrus, which extended into the insula.

Neuroimaging studies in cluster headache have shown that the hypothalamus is likely to be involved during acute cluster headache attacks (May et al., 1998, Sprenger et al., 2004, Morelli et al., 2009, Morelli et al., 2013). Furthermore, there are structural and functional connectivity changes in the pain matrix, with a dynamically altered frontal top-down pain modulation between in bout and out of bout phases (Yang et al., 2018).

A recent functional connectivity study identified reduced functional connectivity from the hypothalamus to the medial frontal cortex in episodic cluster headache patients both ‘in bout’ and ‘out of bout’ compared with healthy controls (Yang et al., 2015). The change in connectivity was observed in the hemisphere contralateral to the attacks. The medial frontal cortex is believed to involve pain anticipation (Ploghaus et al., 1999) and pain modulation through the anterior cingulate cortex. The same authors found reduced grey matter volume in the contralateral medial frontal gyri of episodic cluster headache patients ‘in bout’ compared with healthy controls (Yang et al., 2013). These studies were not performed during cluster headache attacks but interictally during the ‘in bout’ phase; therefore, our observation during acute cluster headache attacks showed increased CBF in the ipsilateral medial frontal cortex, which may indicate pain anticipation and modulation.

For over 100 years, the inferior frontal gyrus was thought to represent motor speech production and was called “Broca’s area”. However, we now know the function of the inferior frontal gyrus is not limited to semantic, phonological processing, but the left inferior frontal gyrus is also involved in working memory and processing of emotional behaviour and empathy (Liakakis et al., 2011). The inferior frontal gyrus also plays a part in the central pain processing network (Tracey, 2008) and is likely to contribute to pain modulation and inhibition. Therefore, the inferior frontal gyrus change during acute cluster headache attack can reflect the lack of top-down pain modulation resulting in extreme pain and an effect on working memory and emotional empathy. Other cluster headache neuroimaging studies have also found changes involving the inferior frontal gyrus (Chou et al., 2017, Qiu et al., 2013, Giorgio et al., 2020). Within this cluster, changes in regional perfusion extended into the insula cortex. The insula has several functional divisions involved in somatosensory, autonomic, interoceptive, salience and cognitive processing (Borsook et al., 2016). The anterior insula has connections to the anterior cingulate cortex, middle and inferior temporal cortices (Cauda et al., 2011). The insula plays a prominent role in pain processing (Brooks and Tracey, 2007) and is often compared to a brain hub (Borsook et al., 2016), where there is a convergence of multiple afferents. Relevant to cluster headache, the insula receives afferent inputs from the thalamus (Noseda et al., 2011) via the trigeminovascular system, an integral part of cluster headache pathophysiology and the pain experienced by cluster headache patients (Goadsby and Edvinsson, 1994, Hoffmann et al., 2019).

The cingulate cortex is involved in the processing of the affective component of pain (Fuchs et al., 2014). Traditionally divided into the anterior cingulate cortex (ACC), midcingulate cortex (MCC) and posterior cingulate cortex; however, imaging studies have demonstrated far more subregions (Vogt, 2014). Negative affect, pain and cognitive control can cause activation in the overlapping regions of the ACC (Shackman et al., 2011).

The clusters of decreased CBF with the highest *T* scores involved the right precuneus, cuneus and superior parietal gyrus, contralateral to the attack side. The precuneus is one of the areas in the default brain network, with high metabolic activity when subjects are at rest, lying quietly and eyes closed (Raichle et al., 2001). Furthermore, it has been suggested that when an individual is aware and alert; however, not engaged in a particular cognitive task; the precuneus is associated with continuously gathering information from the external world (Gusnard et al., 2001). In the case of cluster headache attacks, where the pain experienced by patients has been compared to the pain experienced in gunshot, childbirth and fractures (Burish et al., 2021), it is possible that the pain is so overwhelming that it overrides the default brain network. This may be the case as there is no physiological need to gather information from the surrounding during an attack. Indeed, in a study investigating this phenomenon, the authors found that the default brain network was deactivated when the subjects' attention was maintained on pain (Kucyi et al., 2013).

In the resting-state functional connectivity study previously mentioned, the authors investigated the functional connectivity from the hypothalamus in episodic cluster headache patients in-bout, compared with when they were out-of-bout and with healthy controls (Yang et al., 2015). In their study, images were flipped about the anterior-posterior axis; consequently, all attacks were on the right-side. The authors found that compared with healthy controls, there were functional connectivity changes from the right hypothalamus to the left medial frontal gyrus and right cuneus for episodic cluster headache patients both in- and out-of-bout. They postulated that this change in the ipsilateral cuneus was related to patients experiencing photophobia; however, only seven out of the 18 patients experienced photophobia in their study. Absinta and colleagues found increased grey matter volume in the right cuneus in their tract-based spatial statistics and voxel-based morphometry imaging study comparing 15 episodic cluster headache patients out-of-bout compared with 19 healthy controls (Absinta et al., 2012). None of their cluster headache patients had visual aura; they postulated this reflected the visual system rewiring secondary to the repetition of retro-orbital pain and photophobia during the attacks. In our study, there was reduced CBF in the contralateral cuneus and occipital region; within our cohort, there were four subjects who had migraine with aura, with only subjects 9 and 10 included in the flexible factorial analysis (Table 2) and nine subjects (45%) reported photophobia during their NTG triggered cluster headache attacks. This finding may be due to attention being maintained on the overwhelming pain subjects are experiencing rather than aura or photophobia.

Thus far, the changes seen in CBF are involved in other headache disorders (Messina et al., 2018) and in chronic pain (Tracey and Mantyh, 2007, Lee and Tracey, 2013), therefore from the region of interest analysis, the hypothalamus and ipsilateral ventral pons showed increased CBF during the headache phase are of particular interest. The role of the hypothalamus in cluster headache pathophysiology is strongly supported by the circannual pattern of cluster headache attacks (Kunkle et al., 1952, Kudrow, 1987, Gaul et al., 2012, Lin et al., 2004), neuro-endocrine changes found in cluster headache patients involving the hypothalamus (Leone and Bussone, 1993, Holland and Goadsby, 2009) and previous neuroimaging findings (May et al., 1998, Arkink et al., 2017b, Yang et al., 2015, Ferraro et al., 2018, Sprenger et al., 2004, Morelli et al., 2009, Morelli et al., 2013, Qiu et al., 2013, Sprenger et al., 2006, Qiu et al., 2015). The CBF changes seen within the ventral pons, could reflect changes associated with the trigeminal nerve, which is fundamental in cluster headache pathophysiology, with key roles in the trigeminovascular pathway, trigeminal autonomic reflex and trigeminohypothalamic tracts. Another significant structure involved in cluster headache pathophysiology is the superior salivatory nucleus (SSN), this nucleus is located in the dorsal pons and is vital in the development of the ipsilateral cranial autonomic symptoms cluster headache patients experience during their attacks via activation of the trigeminal autonomic reflex (May and Goadsby, 1999).

The main limitation of the study was that while NTG administration was compared with placebo in cluster headache patients, we did not have an additional arm to compare the effects of NTG alone in healthy subjects or in cluster headache patients who did not develop a cluster headache attack. An investigation of this type would be a valuable way of determining the effects of NTG alone, separately from the effects of headache; however, a PET study demonstrated the areas of increased CBF in NTG spray are the large intracranial vessels, which decreased when subjects developed headache (May et al., 2000). Furthermore, in a 7 Tesla MRI study with healthy volunteers, 0.4 mg sublingual NTG caused middle cerebral artery dilation; however, the overall blood flow did not change; therefore, the authors concluded that although NTG dilates large cerebral arteries, it does not affect the downstream vascular beds (Schulz et al., 2018).

## 5 Conclusions

In conclusion, using a symmetric template and flipping of images in the anterior-posterior axis allows us to superimpose and analyse cluster headache attacks as if they stemmed from symptoms in the same hemisphere with greater confidence in the validity of the inferences. By doing so, we demonstrated increases in CBF in key areas involved in pain anticipation and pain processing. In important regions of interest such as the hypothalamus and ipsilateral ventral pons, we demonstrated significant increases in CBF, reflecting that these structures are important in cluster headache pathophysiology. Lastly, we demonstrate a significant decrease in CBF in the essential areas of the default brain network as attention is shifted due to the severe painful cluster headache attacks that patients are experiencing.

## 6 Ethics statement

This study 16/LO/0693 obtained approval from the London, City & East NHS Research Ethics Committee on the 23^rd^ of June 2016. This study was carried out in accordance with the World Medical Association Declaration of Helsinki (1964), the Research Governance Framework for Health and Social Care (2^nd^ edition, 2005), the Data Protection Act (1998) and the Principles of Good Clinical Practice (GCP), all subjects gave informed consent before taking part.

## CRediT authorship contribution statement

**Diana Y. Wei:** Conceptualization, Methodology, Formal analysis, Investigation, Data curation, Writing – original draft, Writing – review & editing. **Owen O'Daly:** Methodology, Software, Writing – review & editing. **Fernando O. Zelaya:** Methodology, Software, Formal analysis, Supervision, Writing – review & editing. **Peter J. Goadsby:** Conceptualization, Methodology, Supervision, Writing – review & editing.

## Declaration of Competing Interest

The authors declare the following financial interests/personal relationships which may be considered as potential competing interests: DYW no reported competing interests. OOD no reported competing interests. FOZ no reported competing interests. PJG reports, reports, over the last 36 months, grants and personal fees from Amgen and Eli-Lilly and Company, grant from Celgene, and personal fees from Aeon Biopharma, Allergan, Biohaven Pharmaceuticals Inc., Clexio, Electrocore LLC, eNeura, Epalex, GlaxoSmithKline, Impel Neuropharma, Lundbeck, Novartis, Pfizer, Praxis, Sanofi, Santara Therapeutics, Satsuma, and Teva Pharmaceuticals, and personal fees for advice through Gerson Lehrman Group and Guidepoint, fees for educational materials from Massachusetts Medical Society, Medery, Medlink, PrimeEd, UptoDate, WebMD, and publishing royalties from Oxford University Press, and Wolters Kluwer, and for medicolegal advice in headache, and a patent magnetic stimulation for headache (No. WO2016090333 A1) assigned to eNeura without fee.
