# Supplementary material for: Areas of cerebral blood flow changes on arterial spin labelling with the use of symmetric template during nitroglycerin triggered cluster headache attacks
Source: Neuroimage Clin. 2021 Dec 22;33:102920. doi: 10.1016/j.nicl.2021.102920 (PMC8724947; doi:10.1016/j.nicl.2021.102920)
Supplement: Supplementary Data 1 [file mmc1.docx]

Supplementary materials:

| **Non-headache symptoms**  Thirst  Cravings  Yawning  Tiredness  Mood changes  Irritability  Visual blurring  Neck stiffness  Photophobia | Phonophobia  Nausea  Movement sensitivity  Cranial allodynia  Urinary symptoms  Speech difficulties  Concentration difficulties  Gastrointestinal discomfort |
| --- | --- |
| **Cranial autonomic symptoms**  Lacrimation B / R / L  Conjunctival injection B / R / L  Periorbital oedema B / R / L  Itchy/gritty eye B / R / L  Nasal congestion B / R / L  Rhinorrhoea B / R / L  Ptosis B / R / L | Aural fullness B / R / L  Facial flushing B / R / L  Facial swelling B / R / L  Sialorrhoea  Throat tightness  Voice change |
| **Agitation**  Agitation/restlessness |  |

Questionnaire for non-headache and cranial autonomic symptoms. B= bilateral, R= right-sided, L= left-sided.
